# The Pattern of Disposal Practices of Unused and Expired Medications Among Healthcare Professionals: A Cross-Sectional Survey in Rajendra Institute of Medical Sciences, Ranchi, Jharkhand

**DOI:** 10.7759/cureus.27555

**Published:** 2022-08-01

**Authors:** Abha Kumari, Md Shadab Alam, Manisha Kujur, Sandeep Kumar

**Affiliations:** 1 Department of Pharmacology and Therapeutics, Rajendra Institute of Medical Sciences, Ranchi, IND; 2 Department of Preventive and Social Medicine, Rajendra Institute of Medical Sciences, Ranchi, IND; 3 Department of ENT, Rajendra Institute of Medical Sciences, Ranchi, IND

**Keywords:** cross-sectional study, semi-structured questionnaires, health care personnel, disposal practices, unused medications

## Abstract

Background: Medicines play a crucial role in treating various diseases. Most individuals, in their haste to get through their day, have developed the bad habit of improperly discarding their unwanted or expired prescriptions, which poses a number of risks to human health and contributes to environmental degradation. Disposal procedures of unused and expired pharmaceuticals among health care personnel at tertiary care centers have been the subject of very little research to date. Therefore, the purpose of this study is to examine how medical staff at Rajendra Institute of Medical Sciences in Ranchi, Jharkhand, handle stale or unwanted prescription drugs.

Methods: The design of the study was cross-sectional analytical conducted among 385 health care professionals. Pretested semi-structured questionnaires prepared in Google Forms (Google LLC, Mountain View, California, United States) have been used for evaluation.

Results: The completely filled questionnaires were analyzed, evaluated, and expressed in the percentage using Microsoft Excel (Microsoft Corporation, Redmond, Washington, United States). The most common method of disposal of unused and expired medications was throwing them in the dustbin.

Conclusions: In this study, although, all the participants were health care professionals, because of the hectic schedule majority of them used unsafe methods of drug disposal. So, in order to protect our ecosystem, appropriate policy should be made to implement the methods for the safe disposal of unused and expired medications.

## Introduction

The use of medicines for the treatment of various ailments dates back millennia and, with the advent of new techniques and technologies, the need for medicines has been steadily growing worldwide. However, it is equally crucial to properly dispose of any medications that have expired or are no longer needed. Over-the-counter and prescription drug consumption is projected to reach 4.5 trillion doses by 2020, up 24% from 2015 [1]. There are a number of reasons why a patient may not take his or her prescription or over-the-counter medications, including side effects from the drug, recovery from sickness, the medicine's expiry date, and the patient's desire to save the medicine for later usage at home, and patient non-adherence [2-5]. Thus, a significant amount of the pharmaceuticals were either wasted or went out of date [6]. It is crucial for the protection of human health and the natural environment that people understand and follow procedures for safely discarding unused or expired medications. Otherwise, it can lead to various health hazards through poisoning either consumed accidentally or with an intention to commit suicide as well as environmental pollution [7]. The World Health Organization (WHO) recommends taking special care when disposing of pharmaceuticals, prescription medications, patent treatments, vaccinations, and serum that have expired or been used but are no longer needed owing to their chemical or biological nature. Biomedical waste management includes any drugs that have either expired or been abandoned, as defined by Bio-Medical Waste Management (BMW) rule, 2016 [8,9]. The WHO and the Food and Drug Administration (FDA) both have regulations in place regarding the proper disposal of pharmaceuticals. Returning them to the manufacturer, dumping them in a landfill (engineered) after immobilization, incinerating them at medium or high temperatures, chemically decomposing them in accordance with the manufacturer's recommendation, and finally landfilling them are all methods recommended by the WHO for safely disposing of unused or expired medications. The FDA suggests that a "take-back" program is the safest and most effective way to dispose of unwanted medications [10]. Take-back programs are community-based efforts that encourage the general population to surrender unused pharmaceuticals to a designated collection site, where they may then be safely disposed of, rather than turning them over to the authorities as is the case at present.

Disposing of pharmaceutical waste is a big problem for healthcare providers worldwide, particularly in poor nations like India. Very little research has been done so far on the topic of how medical staff at tertiary care facilities dispose of unused and expired pharmaceuticals. Therefore, the purpose of this research is to examine how different types of healthcare workers handle leftover or expired medicine, with the hope of informing policy changes that would ensure these drugs are properly disposed of. The goal of this study is to examine how medical staff at Rajendra Institute of Medical Sciences in Ranchi, Jharkhand, typically dispose of unwanted or out-of-date drugs.

## Materials and methods

The design of the study was a cross-sectional analytical conducted among 385 healthcare professionals that included faculties, resident doctors (both senior and junior residents), medical officers, paramedical staff such as nurses, laboratory technicians, OT assistants, pharmacists, all medical, dental and paramedical students of Rajendra Institute of Medical Sciences, Ranchi. With clearance from the institutional ethics committee of the Rajendra Institute of Medical Sciences, Ranchi, India (memo no. 263 IEC, RIMS, dated 19-06-2021), the research was carried out at that institution's department of pharmacology and therapeutics.

After obtaining appropriate informed permission, the study only included those healthcare providers who were readily accessible and eager to participate, whereas those who had any kind of communication issue or were unwilling to engage were left out of the study. An appropriate calculation was used to determine that 385 people would make up an adequate sample size. A pretested semi-structured questionnaire, prepared in Google Forms (Google LLC, Mountain View, California, United States), was distributed to participants through WhatsApp (Meta Platforms, Inc., Menlo Park, California, United States) or email after obtaining informed permission from each individual.

Participants were briefed about the study's significance and goals before they were asked to fill out the questionnaire. Participants' age, gender, department, and designation were collected, and they were also asked about their typical methods for getting rid of old or unneeded medicine. Participants were then allowed to completely fill out the questionnaires. Finally, the completely filled questionnaire was taken for data analysis.

## Results

A total of 385 participants, which included healthcare professionals, were evaluated regarding the pattern of disposal practices of unused and expired medications. The completely filled questionnaires were analyzed, evaluated, and finally expressed in the percentage using Microsoft Excel (Microsoft Corporation, Redmond, Washington, United States).

Table 1 provides the demographic information for all participants. Seventy-four percent of them are between the ages of 18 and 40, 22.4% are between the ages of 41 and 60, and 3.6% are between the ages of 61 and 70. The response of male participants in this study was 68.8%. As far as ethnicity is concerned, 77.2% belong to a non-tribal group. Since all the participants in this study are healthcare professionals, most of the participants are educated including 61.5% undergraduate medical, dental, and paramedical students, 26.5% graduate, 10.1% postgraduate, and 1.9 % post-doctorate.

**Table 1:** Showing details of participants' demographic data regarding pattern of disposal practices of unused and expired medications among healthcare professionals

S.no	Demographic parameters		Percentage
1.	Age	18-40 years	74%
		41-60 years	22.4%
		61-70 years	3.6%
2.	Gender	Male	68.8%
		Female	31.2%
3.	Education	Undergraduate	61.5%
		Graduate	26.5%
		Postgraduate	10.1%
		Post-doctorate	1.9%
4.	Ethnicity	Tribal	22.8%
		Non-tribal	77.2%

**Table 2:** Reasons for leftover unused and expired medications at home among healthcare professionals

Healthcare professionals	Medical conditions improved	Shift to another treatment	Adverse effects of the drugs	Non-compliance	Excess quantity supplied
Faculties and medical officers	44.6%	28.2%	1.8%	2.6%	22.8%
Senior and junior residents	49%	22.8%	1.2%	18.9%	8.1%
Undergraduate medical and dental students	53%	24%	0.8%	18.2%	4%
Paramedical students	55.5%	23.8%	0.6%	16.7%	3.4%
Paramedical staff	50.8%	21.5%	0.7%	19.8%	7.2%

It is clear from Table 2 that the reason for leftover medication at home for most of the participants, i.e., 44.6% faculties and medical officers, 49% senior and junior residents, 53% undergraduate medical and dental students, 55.5% paramedical students, and 50.8% paramedical staff is the improvement of medical conditions for which the medication was being used. Other factors that are responsible for leftover unused medications at home include adverse side effects of the drugs, non-compliance, excess quantities supplied, and shift to another mode of treatment for early recovery from the illness.

**Figure 1:**
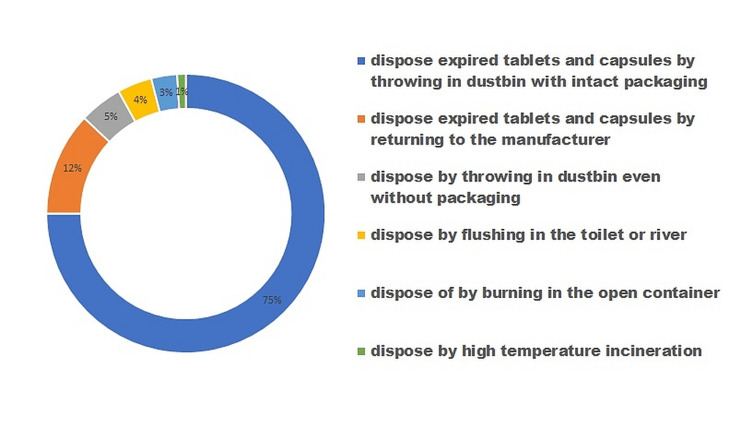
Disposal practices of expired tablets and capsules

Figure 1 depicts that most of the participants (75%) dispose of expired tablets and capsules by throwing them in dustbins with intact packaging, while 5% throw them in dustbins without the packaging. Of the participants, 4% dispose of unused medication by flushing in the toilet or river, 3% dispose by burning in an open container, 1% use high-temperature incineration, and only 12% dispose of expired tablets and capsules by returning to the manufacturer.

Results suggest that 37.1% of the participants dispose of expired injectables by returning them to the manufacturer while 54% of the participants dispose of expired injectables by throwing them in a dustbin with intact packaging, 4.6% in dustbins without the packaging, 1.8% dispose by burning in the open container, and only 2.5% of the participants dispose by high-temperature incineration method.

Similarly, 48% dispose of expired ointment, cream, and lotions by throwing them in dustbins with intact packaging, while 24.6% do the same without the packaging. Of the participants, 17% said that they dispose of expired ointment, cream, and lotions by flushing in the toilet or river and 1.5% dispose by burning in open containers. The method of disposal by high-temperature incineration is used by 1.8% of the participants and only 7.1% of the participants dispose by returning to the manufacturer.

## Discussion

Based on the results of our survey, it is clear that the majority of healthcare workers are choosing dangerous ways of disposing of unwanted and expired pharmaceuticals, which poses a serious problem in the field. 

Table 2 clearly depicts that the main reasons for leftover medications among the participants were early recovery and improvement from the disease, shift to another mode of treatment when not recovered within a short span of time, non-compliance because of a busy life schedule, adverse effects of the drugs and when supplied or purchased in excess of prescribed medications. Figure 1 shows that 75% of respondents reported routinely discarding unused or expired tablets and capsules by placing them in the trash. This percentage is lower than those reported in studies done in Bangladesh (96.8%) [11] and Malaysia (93.6%) [12], but it is higher than that reported in a study done in Harare, Zimbabwe (65%) [13]. Only 4% of participants disposed of expired tablets and capsules by flushing them down the toilet or into a river, while 17% of participants are in the habit of disposing of expired ointment, cream, and lotions this way. This is less than the other study carried out on the same topic, which found that 29% of participants disposed of their unused and expired medications in this way [14]. This distinction may be due to participants' conviction that old or unused medicines should be disposed of by flushing them down the toilet or into a river to avoid unintentional poisoning of children and the environment. However, it has been noted that flushing unneeded drugs causes water pollution and may harm aquatic life. This is because chemicals may reach our waterways after being flushed down the toilet or down a drain, posing a threat to aquatic life in water bodies like lakes and rivers [15]. It has also been clear from earlier studies that pharmaceutical ingredients like various antibiotics, diuretics, mood stabilizers, and antihypertensives present either in groundwater or the wastewater system may be responsible for detrimental health impacts for humans and may cause antibiotics resistance and hormonal imbalance, especially among teenagers [16,17].

In a study by Kuspis and Krenzelok, 1.4% returned medications to a pharmacy, 54% disposed of them in the dustbin, and 35.4% flushed them down the toilet [18]. In our study, all the participants were healthcare professionals; hence, most of the participants especially senior and junior residents as well as paramedical staff have very busy schedules and this may be a cause for non-adherence to disposal directives for the prescribed medications [19]. Appropriate policies should be made to implement safe disposal methods of unused and expired medications.

The primary limitation of this particular study is the number of people studied. We recommend larger studies in the future to come to a better conclusion and also recommend studies on how to curb unsafe methods for disposal.

## Conclusions

When it comes to protecting human health and the natural environment, understanding and the practice of correct disposal of expired or unused drugs are crucial. The participants' preferred method of drug disposal was dumping it in the trash, followed by flushing it down the toilet or washing it away in a river. Some of the participants disposed of expired capsules, tablets, expired injectables, expired ointment, cream, and lotions by burning them in open containers while very few of them disposed of unused or half-used medication by high-temperature incineration. Being healthcare professionals, expired tablets and capsules, injectables and ointment, cream, and lotions should be disposed of by returning to the manufacturer. In order to protect our ecosystem, appropriate policies should be made to implement the methods for the safe disposal of unused and expired medications.
